# Population-based cohort study investigating the association between weight loss and pyogenic liver abscesses

**DOI:** 10.1051/bmdcn/2017070426

**Published:** 2017-11-24

**Authors:** Shih-Wei Lai, Cheng-Li Lin, Kuan-Fu Liao

**Affiliations:** 1 College of Medicine, China Medical University Taichung 404 Taiwan; 2 Department of Family Medicine, China Medical University Hospital Taichung 404 Taiwan; 3 Management Office for Health Data, China Medical University Hospital Taichung 404 Taiwan; 4 College of Medicine, Tzu Chi University Hualien 970 Taiwan; 5 Department of Internal Medicine, Taichung Tzu Chi General Hospital Taichung 427 Taiwan; 6 Graduate Institute of Integrated Medicine, China Medical University Taichung 404 Taiwan

**Keywords:** Weight loss, Alcohol-related disease, Biliary stone, Diabetes mellitus, Pyogenic liver abscesses

## Abstract

Background and Aim: Few systematic studies focus on the association between weight loss and pyogenic liver abscesses. The objective of the study was to assess the association between weight loss and pyogenic liver abscesses in adults in Taiwan.

Methods: This population-based cohort study utilized the database of the Taiwan National Health Insurance Program. Totally, 8453 subjects aged 20 to 84 years with newly diagnosed weight loss between 2000 and 2012 were assigned as the weight loss group, and 33777 randomly selected subjects without weight loss were assigned as the non-weight loss group. Both the weight loss and the non-weight loss groups were matched according to sex, age, and comorbidities. The incidence of pyogenic liver abscesses at the end of 2013 was measured in both groups.

Results: A multivariable Cox proportional hazards regression model was done and presented evidence that the adjusted HR of pyogenic liver abscess was 2.47 (95 %CI 1.21, 5.02) for those subjects with weight loss and without comorbidities, as compared with those subjects without weight loss and without comorbidities. Among the weight loss group, 5% developed pyogenic liver abscesses within 3 months.

Conclusion: Weight loss is associated with pyogenic liver abscesses in adults. Yet weight loss might not be an early clinical symptom of undiagnosed pyogenic liver abscesses.

## 1. Introduction

A pyogenic liver abscess is a liver infection which is caused by a broad range of microorganisms. Pyogenic liver abscesses are major public health concern due to their significant morbidity and mortality. Their mortality rate was around 2.5% to 31% in previous studies, depending on the study year and the study population [1, 2] Growing evidence has shown that a variety of precipitating factors are associated with pyogenic liver abscesses, including biliary tract disease, diabetics mellitus, end-stage renal disease, splenectomy, herpes zoster, and cancer. [3-8] The previous case-series studies have shown that the most common presenting symptoms of pyogenic liver abscesses are fever and right upper quadrant pain. [7, 9-14]

Weight loss is usually defined as a loss of 5% or more of one’s baseline body weight within 6 months. [15-17] Weight loss is a common complaint frequently associated with a wide variety of diseases. The literature has shown that gastrointestinal disorders, psychiatric disorders, and cancers are usually the most prevalent causes of weight loss. [17-19] Some case-series studies have shown that weight loss can be found in patients with pyogenic liver abscesses. [11-14] However, no study has been done based on a systematic analysis that examines the association between weight loss and pyogenic liver abscesses. If a well-designed study could establish that weight loss might be an early clinical symptom of an undiagnosed pyogenic liver abscess, clinicians could consider the possibility of a pyogenic liver abscess when a patient presents with weight loss accompanied by fever and right upper quadrant pain. Therefore, we conducted a population-based cohort study to investigate the association between weight loss and pyogenic liver abscesses in adults in Taiwan.

## 2. Methods

### 2.1. Design and data source

We designed a population-based cohort study utilizing the data base of the Taiwan National Health Insurance Program. Briefly, Taiwan is an independent country with a population of more than 23 million people. [20-30] This insurance program began on March 1st, 1995, and the enrollment rate was over 99.6% of the 23 million people living in Taiwan by the end of 2015. [31] The details of the program have been well reported in previous studies. [32-36] The present study was approved by the Research Ethics Committee of China Medical University and Hospital in Taiwan (CMUH-104-REC2-115).

### 2.2. Participants and inclusion criteria

**Fig. 1:**
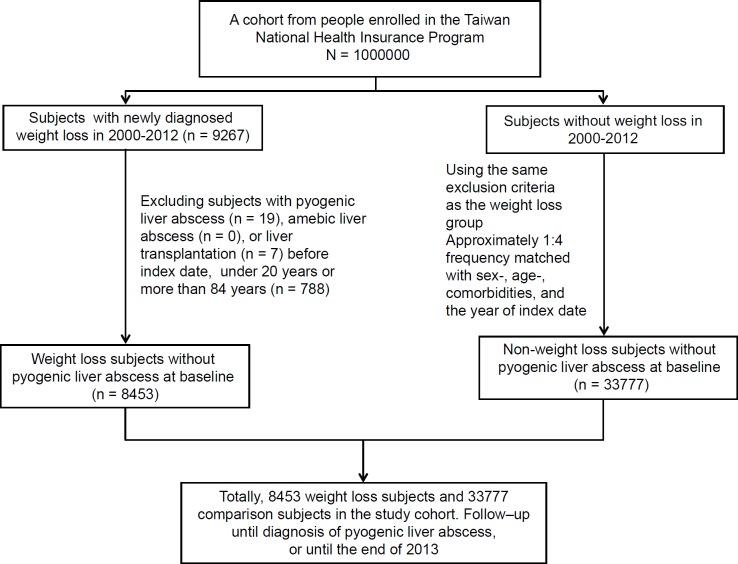
-Flow chart showing the selection process for the study’s subjects.

Based on the International Classification of Diseases 9th Revision-Clinical Modification (ICD-9 code), the weight loss group for this study was defined as the subjects aged 20 to 84 years with newly diagnosed weight loss between 2000 and 2012 (ICD-9 code 783.21). The index date was defined as the date of being diagnosed with weight loss. For each subject diagnosed with weight loss that was selected, approximately 4 subjects without weight loss were randomly selected from the same database as the non-weight loss group. Both the weight loss and the non-weight loss groups were matched by sex, age (within 5 years), comorbidities, and the year of index date. Subjects who had a history of pyo-genic liver abscess, amebic liver abscess, or liver transplantation before the index date were excluded from the study (Fig. 1).

### 2.3. Comorbidities potentially associated with pyogenic liver abscess

Comorbidities before the index date that were included are as follows: alcohol-related disease, biliary stone, chronic kidney disease, chronic liver disease (including cirrhosis, hepatitis B infection, hepatitis C infection, and other chronic hepatitis), and diabetes mellitus. The diagnostic correctness of these comorbidities based on ICD-9 codes has been well assessed in previous studies. [37-46]

### 2.4. Major outcome

The major outcome was a new diagnosis of pyogenic liver abscess (ICD-9 code 572.0) according to a discharge diagnosis of hospitalization during the follow-up period. All study subjects were followed until either they were diagnosed with pyogenic liver abscesses or until the end of 2013.

### 2.5. Statistical analysis

The distributions of sex, age, and comorbidities were compared between the weight loss group and the non-weight loss group using a *Chi*-square test for categorized variables and a t-test for continuous variables. The incidence of pyogenic liver abscesses was measured as the event number of pyogenic liver abscesses identified during the follow-up period, divided by the total follow-up person-years for each group. Initially, all variables were included in a univariable model. Next, variables found to be statistically significant in the univariable model were further included in the multivariable model. A multivariable Cox proportional hazards regression model was used to assess the hazard ratio (HR) and 95 % confidence interval (CI) for the risk of having a pyogenic liver abscess associated with weight loss and comorbidities including biliary stone, chronic liver disease, and diabetes mellitus. All analyses were performed with SAS software version 9.2 (SAS Institute Inc., Cary, North Carolina, USA). Two-tailed *P* < 0.05 was considered statistically significant.

## 3. Results

### 3.1. Demographic characteristics of the study population

Table 1 presents the distributions of sex, age, and comorbidities between the weight loss group and the non-weight loss group. In total, 8453 subjects in the weight loss group and 33777 subjects in the non-weight loss group were selected, with similar distributions of sex and age between the two groups. The mean ages (standard deviation) of the study subjects were 54.6 (16.4) years for the weight loss group and 54.3 (16.5) years for the non-weight loss group (*t*-test, *P* = 0.08). The proportions of alcohol-related disease, biliary stone, chronic kidney disease, chronic liver disease, and diabetes mellitus were equally distributed in the weight loss group and the non-weight loss group (*Chi*-square test, *P* > 0.05 for all).

**Table 1:** Baseline characteristics between the weight loss and the non-weight loss groups.

	Weight loss N = 8453		Non-weight loss N = 33777		
**Variable**	n	(%)	n	(%)	*P* value*
Sex					0.99
Female	4012	(47.5)	16028	(47.5)	
Male	4441	(52.5)	17749	(52.5)	
Age group (years)					0.95
20-39	1811	(21.4)	7287	(21.6)	
40-64	4139	(49.0)	16490	(48.8)	
65-84	2503	(29.6)	10000	(29.6)	
Age (years), mean (standard deviation)†	54.6	(16.4)	54.3	(16.5)	0.08
Baseline comorbidities					
Alcohol-related disease	691	(8.17)	2744	(8.12)	0.88
Biliary stone	276	(3.27)	1078	(3.19)	0.73
Chronic kidney disease	302	(3.57)	1181	(3.50)	0.73
Chronic liver disease	2330	(27.6)	9298	(27.5)	0.95
Diabetes mellitus	704	(8.33)	2799	(8.29)	0.90

Data are presented as the number of subjects in each group with percentages given in parentheses, or mean with standard deviation given in parentheses.

* *Chi*-square test,

† *t* test comparing subjects with and without weight loss.

### 3.2. Incidence of pyogenic liver abscesses in the study population stratified by sex, age, and follow-up period

The follow-up results from this study present that the overall incidence of pyogenic liver abscesses was 1.26-fold greater in the weight loss group than in the non-weight loss group (5.27 vs. 4.19 per 10000 person-years, 95% CI 1.15, 1.37) (Table 2). The incidence of pyogenic liver abscesses was higher in male subjects than in female subjects among both the weight loss group and the non-weight loss group. The analysis stratified by follow-up period presents that the incidence of pyogenic liver abscesses was higher after 3 months of diagnosing weight loss (5.30 per 10000 person-years, Table 2). Only 1 patient in the weight loss group (5%, 1/20) developed a pyogenic liver abscess within 3 months. The other 19 patients in the weight loss group (95%) developed pyogenic liver abscesses after 3 months of being diagnosed with weight loss.

**Table 2:** Incidence of pyogenic liver abscesses between weight loss and non-weight loss groups stratified

**Variable**	Weight loss				Non-weight loss				
	N	Event	Person-years	Incidence†	N	Event	Person-years	Incidence†	IRR* (95% CI)
All	8453	20	37929	5.27	33777	67	159763	4.19	1.26 (1.15, 1.37)
Sex									
Female	4012	4	18662	2.14	16028	26	76711	3.39	0.63 (0.54, 0.74)
Male	4441	16	19267	8.30	17749	41	83052	4.94	1.68 (1.51, 1.88)
Age group (years)									
20-39	1811	1	8987	1.11	7287	4	36548	1.09	1.02 (0.82, 1.27)
40-64	4139	12	19013	6.31	16490	26	78554	3.31	1.91 (1.70, 2.13)
65-84	2503	7	9929	7.05	10000	37	44660	8.28	0.85 (0.72, 1.01)
Follow-up period (months)									
<3	8453	1	2095	4.77	33777	2	8430	2.37	2.01 (1.83, 2.22)
≥3	8293	19	35834	5.30	33657	65	151333	4.30	1.23 (1.13, 1.35)

† Incidence: per 10000 person-years.

* IRR (incidence rate ratio): weight loss vs. non-weight loss. (95% confidence interval)

### 3.3. Pyogenic liver abscesses associated with weight loss and comorbidities

Variables found to be statistically significant in the univariable model were further analyzed. Sex, age, biliary stone, chronic liver disease, and diabetes mellitus were compatible with the criteria for adjustment. Finally, biliary stone, chronic liver disease, and diabetes mellitus were included as comorbidities. The multivariable Cox proportional hazards regression model shows that the adjusted HR of pyogenic liver abscesses was 2.47 (95 %CI 1.21, 5.02) for subjects with weight loss and without comorbidities, as compared to subjects without weight loss and without comorbidities (Table 3).

**Table 3:** Cox proportional hazards regression analysis for risk of pyogenic liver abscesses associated with weight loss and comorbidities.

Variable		Event		Person-years		Incidence†		Adjusted HR* (95% CI)
Weight loss	Any comorbidity&						
No	No	21		105925		1.98		(Reference)
No	Yes	46		53838		8.54		3.52 (2.10, 5.91)
Yes	No	12		25193		4.76		2.47 (1.21, 5.02)
Yes	Yes	8		12736		6.28		2.62 (1.16, 5.93)

† Incidence rate: per 10000 person-years.

* Variables found to be statistically significant in the univariable model were included for further analysis. Adjusted for sex and age

& Comorbidities include biliary stone, chronic liver disease, and diabetes mellitus.

## 4. Discussion

In this retrospective cohort study, we observed that the overall incidence of pyogenic liver abscesses was 1.26-fold greater in the weight loss group than in the non-weight loss group. We also observed that patients with weight loss and without comorbidities were associated with a 2.47-fold increased risk of pyogenic liver abscesses, as compared with subjects without weight loss and without comorbidities. In the present study, we selected patients with weight loss prior to a confirmed diagnosis of pyogenic liver abscesses. Although a causal-effect relationship can not be established based on an observational study such as this, weight loss can be said to be substantially associated with pyogenic liver abscesses in adults.

Weight loss is a common clinical problem and its serious underlying disorders are frequently associated with increased morbidity and mortality. Some case-series studies have shown that weight loss is found in patients with pyogenic liver abscesses,[11-14] but these studies did not investigate the percentage of weight loss among patients who had pyogenic liver abscesses, and they did not show the interval between weight loss and pyogenic liver abscesses. Thus, based on those studies we cannot be sure whether weight loss is an early clinical symptom of undiagnosed pyogenic liver abscesses, or weight loss is only an accompanied symptom of coexisting conditions that might predispose someone to have a potential risk for developing a pyogenic liver abscess.

In the present study, we observed that the incidence of pyogenic liver abscesses was higher after 3 months of diagnosing weight loss (5.30 per 10000 person-years, Table 2). Only 5% of patients with weight loss (1/20) developed pyogenic liver abscesses within 3 months, whereas the rest (95%) developed pyogenic liver abscesses after 3 months of being diagnosed with weight loss. Previous case-series studies have shown that the duration from symptoms onset to the patient presenting to a medical facility was around 9-17 days.[9, 11, 12] In view of the high accessibility to medical facilities in Taiwan, if weight loss is really an early clinical symptom of an undiagnosed pyogenic liver abscess, it does not need to take 3 months to make a diagnosis of a pyogenic liver abscess after a patient presents with weight loss to a medical facility. Nevertheless, we think that weight loss may not be an early clinical symptom of undiagnosed pyogenic liver abscesses, but it could be an accompanied symptom of a number of unfound underlying disorders. Fever and right upper quadrant pain are still the chief symptoms of pyogenic liver abscess. [7, 9-14]

Because no other systematic study focuses on this issue, we have tried to make a rational explanation for the association of weight loss with pyogenic liver abscesses, and it is as follows. Initially, patients who present with weight loss might seek consultation for the underlying disorders associated with weight loss. Meanwhile, some of these underlying disorders could be also a risk factor for pyogenic liver abscesses. As time progresses, patients who had this kind of underlying disorder might be more likely to develop pyogenic liver abscesses later. For example, weight loss is an early and typical symptom associated with undiagnosed diabetes mellitus, and diabetes mellitus is well-known to be a risk factor for pyogenic liver abscesses.[5-8] Therefore, if patients who present with weight loss actually have diabetes mellitus, they are also at an increased risk for developing pyogenic liver abscesses later. This explanation can be partially proven by the fact that among the subjects with weight loss and without comorbidities at the baseline (Table 3), 1 subject developed diabetes mellitus, 4 subjects developed cancer, and 2 subjects developed diabetes mellitus and cancer during the follow-up period. As is well-known, diabetes mellitus and cancer are two comorbid conditions associated with an increased risk for developing pyogenic liver abscesses,[5-8] and diabetes mellitus and cancer can cause weight loss. Therefore, it is the underlying disorders associated with weight loss that further increase the risk of pyogenic liver abscesses, not the weight loss itself.

## 5. Limitations

There were some important limitations in the study. First, due to an inherent limitation of the database used, there was no record of whether weight loss was involuntary or voluntary. However, patients who called medical care for weight loss can be considered to be those looking for an underlying disorder that is causing the weight loss. Thus, we think that weight loss of the patients in the database is largely if not entirerly involuntary. Second, due to the same limitation, we could not know what percentage of weight loss was detected when patients presented to medical facilities. ICD-9 code of weight loss could be used instead. Third, due to the same limitation, we did not know whether patients with weight loss also presented with fever or right upper quadrant pain during the same medical visit. That is, we could not determine the chief symptoms among subjects with pyogenic liver abscesses in this study. Fourth, the event number of pyogenic liver abscesses was too small to be convincing as to the statistical significance of this study (only 20 events in the weight loss group and 67 events in the non-weight loss group). More large-size studies with a much larger event number are needed to clarify this issue.

Despite the above limitations, some strengths of this study should be mentioned. This is the first cohort study to link weight loss with pyogenic liver abscesses in adults. The outcome is very difficult to assess as it is not common, but the Taiwan National Health Insurance Program provides an accessible database to explore this association in more depth. The results are relatively impressive. The present study also provides important information on what risks of developing a pyogenic liver abscess should be followed up in Taiwan.

## 6. Conclusion

Weight loss is associated with pyogenic liver abscesses in adults in Taiwan. Among those that developed pyogenic liver abscesses in the weight loss group, only 5% occurred within 3 months. Thus, weight loss might not be an early clinical symptom of an undiagnosed pyogenic liver abscess, but patients with weight loss could be at an increased risk for developing pyogenic liver abscesses later. Clinicians should keep in mind that the underlying disorder associated with weight loss could be a risk factor for pyogenic liver abscesses. We suggest that clinicians not only record the term “weight loss” when recording a patient’s history, but they also should measure body weight every visit. Only serial measurements of body weight can correctly quantify weight loss in clinical practice.

## Specific author contributions

Shih-Wei Lai planned and conducted this study. He contributed to the conception of the article, initiated the draft of the article, and revised the article.

Cheng-Li Lin conducted the data analysis and revised the article.

Kuan-Fu Liao planned and conducted this study. He participated in the data interpretation and revised the article

## Conflict of Interest Statement

The authors wish to disclose no conflicts of interest.

## References

[1] Huang CJ, Pitt HA, Lipsett PA, Osterman FA Jr., Lillemoe KD, Cameron JL, et al Pyogenic hepatic abscess. Changing trends over 42 years. Ann Surg. 1996; 223: 600-7. 8651751 10.1097/00000658-199605000-00016 PMC1235191

[2] Rahimian J, Wilson T, Oram V, Holzman RS. Pyogenic liver abscess: recent trends in etiology and mortality. Clin Infect Dis. 2004; 39: 1654-9. 15578367 10.1086/425616

[3] Tsai LW, Chao PW, Ou SM, Chen YT, Shih CJ, Li SY, et al Pyogenic liver abscess in end-stage renal disease patients: a nationwide longitudinal study. Hemodial Int. 2015; 19: 72-9. 24947911 10.1111/hdi.12185

[4] Chu KM, Fan ST, Lai EC, Lo CM, Wong J. Pyogenic liver abscess. An audit of experience over the past decade. Arch Surg. 1996; 131: 148-52. 8611070 10.1001/archsurg.1996.01430140038009

[5] Shen ML, Liao KF, Tsai SM, Lin CL, Lai SW. Herpes zoster correlates with pyogenic liver abscesses in Taiwan. Biomedicine-Taiwan. 2016; 6: 24-9. 10.7603/s40681-016-0022-4 PMC5112182 27854050

[6] Lai SW, Lai HC, Lin CL, Liao KF. Splenectomy Correlates With Increased Risk of Pyogenic Liver Abscess: A Nationwide Cohort Study in Taiwan. J Epidemiol. 2015; 25: 561-6. 26256773 10.2188/jea.JE20140267 PMC4549607

[7] Chan KS, Chen CM, Cheng KC, Hou CC, Lin HJ, Yu WL. Pyogenic liver abscess: a retrospective analysis of 107 patients during a 3-year period. Jpn J Infect Dis. 2005; 58: 366-8. 16377869

[8] Kaplan GG, Gregson DB, Laupland KB. Population-based study of the epidemiology of and the risk factors for pyogenic liver abscess. Clin Gastroenterol Hepatol. 2004; 2: 1032-8. 15551257 10.1016/s1542-3565(04)00459-8

[9] Bosanko NC, Chauhan A, Brookes M, Moss M, Wilson PG. Presentations of pyogenic liver abscess in one UK centre over a 15-year period. J R Coll Physicians Edinb. 2011; 41: 13-7. 21365060 10.4997/JRCPE.2011.104

[10] Lok KH, Li KF, Li KK, Szeto ML. Pyogenic liver abscess: clinical profile, microbiological characteristics, and management in a Hong Kong hospital. J Microbiol Immunol Infect. 2008; 41: 483-90. 19255692

[11] Hernandez JL, Ramos C. Pyogenic hepatic abscess: clues for diagnosis in the emergency room. Clin Microbiol Infect. 2001; 7: 567-70. 11683800 10.1046/j.1198-743x.2001.00323.x

[12] Santos-Rosa OM, Lunardelli HS, Ribeiro-Junior MA. Pyogenic Liver Abscess: Diagnostic and Therapeutic Management. Arq Bras Cir Dig. 2016; 29: 194-7. 27759785 10.1590/0102-6720201600030015 PMC5074673

[13] Islam Q, Ekram A, Ahmed M, Alim M, Ahad M, Haque M, et al Pyogenic liver abscess and indigenous alcohol. TAJ: Journal of Teachers Association. 2005; 18: 21-4.

[14] Ghosh S, Sharma S, Gadpayle AK, Gupta HK, Mahajan RK, Sahoo R, et al Clinical, Laboratory, and Management Profile in Patients of Liver Abscess from Northern India. Journal of Tropical Medicine. 2014; 2014: 8. 10.1155/2014/142382 PMC4066852 25002869

[15] Sahyoun NR, Serdula MK, Galuska DA, Zhang XL, Pamuk ER. The epidemiology of recent involuntary weight loss in the United States population. J Nutr Health Aging. 2004; 8: 510-7. 15543425

[16] Moriguti JC, Moriguti EK, Ferriolli E, de Castilho Cacao J, Iucif N Jr., Marchini JS. Involuntary weight loss in elderly individuals: assessment and treatment. Sao Paulo Med J. 2001; 119: 72-7. 11276170 10.1590/S1516-31802001000200007 PMC11159575

[17] Wong CJ. Involuntary weight loss. Med Clin North Am. 2014; 98: 625-43. 24758965 10.1016/j.mcna.2014.01.012

[18] Alibhai SM, Greenwood C, Payette H. An approach to the management of unintentional weight loss in elderly people. Cmaj. 2005; 172: 773-80. 15767612 10.1503/cmaj.1031527 PMC552892

[19] Baicus C, Caraiola S, Baicus A, Tanasescu R, Rimbas M. Involuntary weight loss: case series, etiology and diagnostic. Rom J Intern Med. 2009; 47: 87-92. 19886074

[20] Yang MD, Lin KC, Lu MC, Jeng LB, Hsiao CL, Yueh TC, et al Contribution of matrix metalloproteinases-1 genotypes to gastric cancer susceptibility in Taiwan. Biomedicine-Taiwan. 2017; 7: 18-24. 10.1051/bmdcn/2017070203 PMC5479427 28612708

[21] Yang JS, Lu CC, Kuo SC, Hsu YM, Tsai SC, Chen SY, et al Autophagy and its link to type II diabetes mellitus. Biomedicine-Taiwan. 2017; 7: 1-12. 10.1051/bmdcn/2017070201 PMC5479440 28612706

[22] Wu MH, Lee TH, Lee HP, Li TM, Lee IT, Shieh PC, et al Kuei-Lu-Er-Xian-Jiao extract enhances BMP-2 production in osteoblasts. Biomedicine-Taiwan. 2017; 7: 9-15. 10.1051/bmdcn/2017070102 PMC5439337 28474578

[23] Liu YL, Liu JH, Wang IK, Ju SW, Yu TM, Chen IR, et al Association of inflammatory cytokines with mortality in peritoneal dialysis patients. Biomedicine-Taiwan. 2017; 7: 1-8. 10.1051/bmdcn/2017070101 PMC5439334 28474577

[24] Lin CH, Lin WC, Chang JS. Presentations and management of different causes of chylothorax in children: one medical center’s experience. Biomedicine-Taiwan. 2017; 7: 30-4. 10.1051/bmdcn/2017070105 PMC5439341 28474581

[25] Liao CF, Yang TY, Chen YH, Yao CH, Way TD, Chen YS. Effects of swimming exercise on nerve regeneration in a rat sciatic nerve transection model. Biomedicine-Taiwan. 2017; 7: 16-24. 10.1051/bmdcn/2017070103 PMC5439339 28474579

[26] Lin HF, Lai SW, Lin WY, Liu CS, Lin CC, Chang CM. Prevalence and factors of elevated alanine aminotransferase in central Taiwan-a retrospective study. Biomedicine-Taiwan. 2016; 6: 25-30. 10.7603/s40681-016-0011-7 PMC4864771 27161001

[27] Lai SW. Risks and benefits of zolpidem use in Taiwan: a narrative review. Biomedicine-Taiwan. 2016; 6: 9-11. 10.7603/s40681-016-0008-2 PMC4859316 27154196

[28] Cheng KC, Lin WY, Liu CS, Lin CC, Lai HC, Lai SW. Association of different types of liver disease with demographic and clinical factors. Biomedicine-Taiwan. 2016; 6: 16-22. 10.7603/s40681-016-0016-2 PMC4996334 27518399

[29] Cheng KC, Lee TL, Lin YJ, Liu CS, Lin CC, Lai SW. Facility evaluation of resigned hospital physicians:managerial implications for hospital physician manpower. Biomedicine. 2016; 6: 30-9. 10.7603/s40681-016-0023-3 PMC5112183 27854049

[30] Chen CM, Lai CH, Wu HJ, Wu LT. Genetic characteristic of class 1 integrons in proteus mirabilis isolates from urine samples. Biomedicine-Taiwan. 2017; 7: 12-7. 10.1051/bmdcn/2017070202 PMC5479437 28612707

[31] Ministry of Health and Welfare Taiwan. 2016 Taiwan Health and Welfare Report. http://www.mohw.gov.tw. [cited on July 1, 2017, English version].

[32] Lai SW, Liao KF, Liao CC, Muo CH, Liu CS, Sung FC. Polypharmacy correlates with increased risk for hip fracture in the elderly: a population-based study. Medicine. 2010; 89: 295-9. 20827106 10.1097/MD.0b013e3181f15efc

[33] Hung SC, Lai SW, Tsai PY, Chen PC, Wu HC, Lin WH, et al Synergistic interaction of benign prostatic hyperplasia and prostatitis on prostate cancer risk. Br J Cancer. 2013; 108: 1778-83. 23612451 10.1038/bjc.2013.184 PMC3658521

[34] Liao KF, Lai SW, Li CI, Chen WC. Diabetes mellitus correlates with increased risk of pancreatic cancer: a population-based cohort study in Taiwan. J Gastroenterol Hepatol. 2012; 27: 709-13. 21929650 10.1111/j.1440-1746.2011.06938.x

[35] Lai HC, Tsai IJ, Chen PC, Muo CH, Chou JW, Peng CY, et al Gallstones, a cholecystectomy, chronic pancreatitis, and the risk of subsequent pancreatic cancer in diabetic patients: a population-based cohort study. J Gastroenterol. 2013; 48: 721-7. 23053420 10.1007/s00535-012-0674-0

[36] Chen HY, Lin CL, Lai SW, Kao CH. Association of Selective Serotonin Reuptake Inhibitor Use and Acute Angle-Closure Glaucoma. J Clin Psychiatry. 2016; 77: e692-6. 27135704 10.4088/JCP.15m10038

[37] Lai SW, Liao KF, Lin CL, Chen PC. Pyogenic liver abscess correlates with increased risk of acute pancreatitis: a population-based cohort study. J Epidemiol. 2015; 25: 246-53. 25716281 10.2188/jea.JE20140152 PMC4341002

[38] Liao KF, Lai SW, Lin CL, Chien SH. Appendectomy correlates with increased risk of pyogenic liver abscess: A population-based cohort study in Taiwan. Medicine. 2016; 95: e4015. 27368018 10.1097/MD.0000000000004015 PMC4937932

[39] Lai SW, Lin CL, Liao KF. Rheumatoid Arthritis and Risk of Pyogenic Liver Abscess in Taiwan. International Medical Journal. 2016; 23: 267-8.

[40] Liao KF, Cheng KC, Lin CL, Lai SW. Etodolac and the risk of acute pancreatitis. Biomedicine-Taiwan. 2017; 7: 25-9. 10.1051/bmdcn/2017070104 PMC5439338 28474580

[41] Liao KF, Lin CL, Lai SW, Chen WC. Zolpidem Use Associated With Increased Risk of Pyogenic Liver Abscess: A Case-Control Study in Taiwan. Medicine. 2015; 94: e1302. 26266369 10.1097/MD.0000000000001302 PMC4616684

[42] Lai SW, Lin CL, Liao KF. Atrial fibrillation associated with acute pancreatitis: a retrospective cohort study in Taiwan. J Hepatobiliary Pancreat Sci. 2016; 23: 242-7. 26842603 10.1002/jhbp.331

[43] Liao KF, Cheng KC, Lin CL, Lai SW. Statin Use Correlates with Reduced Risk of Pyogenic Liver Abscess: A Population-Based Case-Control Study. Basic Clin Pharmacol Toxicol. 2017; 121: 144-9. 28273396 10.1111/bcpt.12777

[44] Lin HF, Liao KF, Chang CM, Lin CL, Lai SW. Correlation between proton pump inhibitors and risk of pyogenic liver abscess. Eur J Clin Pharmacol. 2017; 73: 1019-25. 28434021 10.1007/s00228-017-2256-9

[45] Liao KF, Huang PT, Lin CC, Lin CL, Lai SW. Fluvastatin use and risk of acute pancreatitis:a population-based case-control study in Taiwan. Biomedicine-Taiwan. 2017; 7: 24-8. 10.1051/bmdcn/2017070317 PMC5571662 28840831

[46] Lai SW, Lin CL, Liao KF. Risk of contracting pneumonia among patients with predialysis chronic kidney disease: a population-based cohort study in Taiwan. Biomedicine-Taiwan. 2017; 7: 42-7. 10.1051/bmdcn/2017070320 PMC5571660 28840834

